# Supplementary material for: Anti-Ku Antibodies: Clinical Associations, Organ Damage, and Prognostic Implications in Connective Tissue Diseases
Source: Int J Mol Sci. 2025 Aug 1;26(15):7433. doi: 10.3390/ijms26157433 (PMC12347976; doi:10.3390/ijms26157433)
Supplement: Supplementary file 1 [file ijms-26-07433-s001.zip › ijms-3771883-supplementary.pdf]

Supplementary Table S1 – Clinical and biological features of the anti-Ku positive patients

Clinical and biological parameters	Value
Arthralgia	43/46 (93.5)
Arthritis	19/46 (41.3)
Raynaud	10/24 (41.7)
Muscle weakness	15/45 (33.3)
Dysphagia	2/43 (4.7)
Serositis	6/42 (14.3)
Lupus rash	8/41 (19.5)
Photosensitivity	7/30 (23.3)
Thrombosis	4/45 (8.9)
Neuropathy	12/22 (54.5)
Sicca syndrome	21/31 (67.7)
Myocarditis	5/44 (11.4)
Vasculitis	4/40 (10.0)
Lymphoma	2/47 (4.3)
Cryoglogulinemia	1/13 (7.7)
Elevated CK	12/44 (27.3)
Cytopenia	13/47 (27.7)
Low C3	7/31 (22.6)
Low C4	5/31 (16.1)
Elevated dsDNA	9/45 (20.0)
SSA	20/47 (42.6)
Ro60	13/27 (48.1)
Ro52	15/29 (51.7)
SSB	4/47 (8.5)
Sm	4/47 (8.5)
RNP	6/47 (12.8)
Mi2	5/47 (10.6)
PMScl75	3/13 (23.1)
PMScl100	1/12 (8.3)
PL12	4/14 (28.6)
SRP	1/6 (16.7)
RF	7/37 (18.9)
ACPA	2/28 (7.1)
Antiphospholipid biology	11/24 (45.8)
ANCA	4/33 (12.1)

Data are shown as n/N (%). CK, creatine kinase; dsDNA, double-stranded DNA.
